# [Corrigendum] Overexpression of SET oncoprotein is associated with tumor progression and poor prognosis in human gastric cancer 

**DOI:** 10.3892/or.2025.8976

**Published:** 2025-08-21

**Authors:** Xiaoning Yuan, Te Zhang, Xin Zheng, Yunfei Zhang, Tingting Feng, Pengfei Liu, Zhiting Sun, Shanshan Qin, Xuewen Liu, Liang Zhang, Jie Song, Ying Liu

Oncol Rep 38: 1733–1741, 2017; DOI: 10.3892/or.2017.5788

Subsequently to the publication of the above paper, the authors contacted the Editor to explain that, concerning the cell invasion assay experiments shown in Fig. 4, and due to an error made in filing the data, the image featured for the OA group / MGC803 cellular experiment in Fig. 4C had been inadvertently duplicated from the invasion image showing the shSET group / SGC7901 cellullar experiment in Fig. 1A. Additionally, a partial overlap was noted between the migration image of the MGC803 cells for the NC siRNA group in Fig. 4D (scratch-wound assay experiments) and the corresponding image in Fig. 3B. Although both images represent the same cell line with the NC siRNA group, the authors wished to provide a revised version of this data panel in Fig. 4D (in addition to the correction needed for Fig. 4C) for the sake of clarity.

The corrected version of Fig. 4 is shown on the next page. Note that the revisions made to this figure do not affect the overall conclusions reported in the paper, and all the authors agree with the publication of this corrigendum. The authors are grateful to the Editor of *Oncology Reports* for allowing them the opportunity to publish this Corrigendum; furthermore, they apologize to the readership for any inconvenience caused.

## Figures and Tables

**Figure 4.**
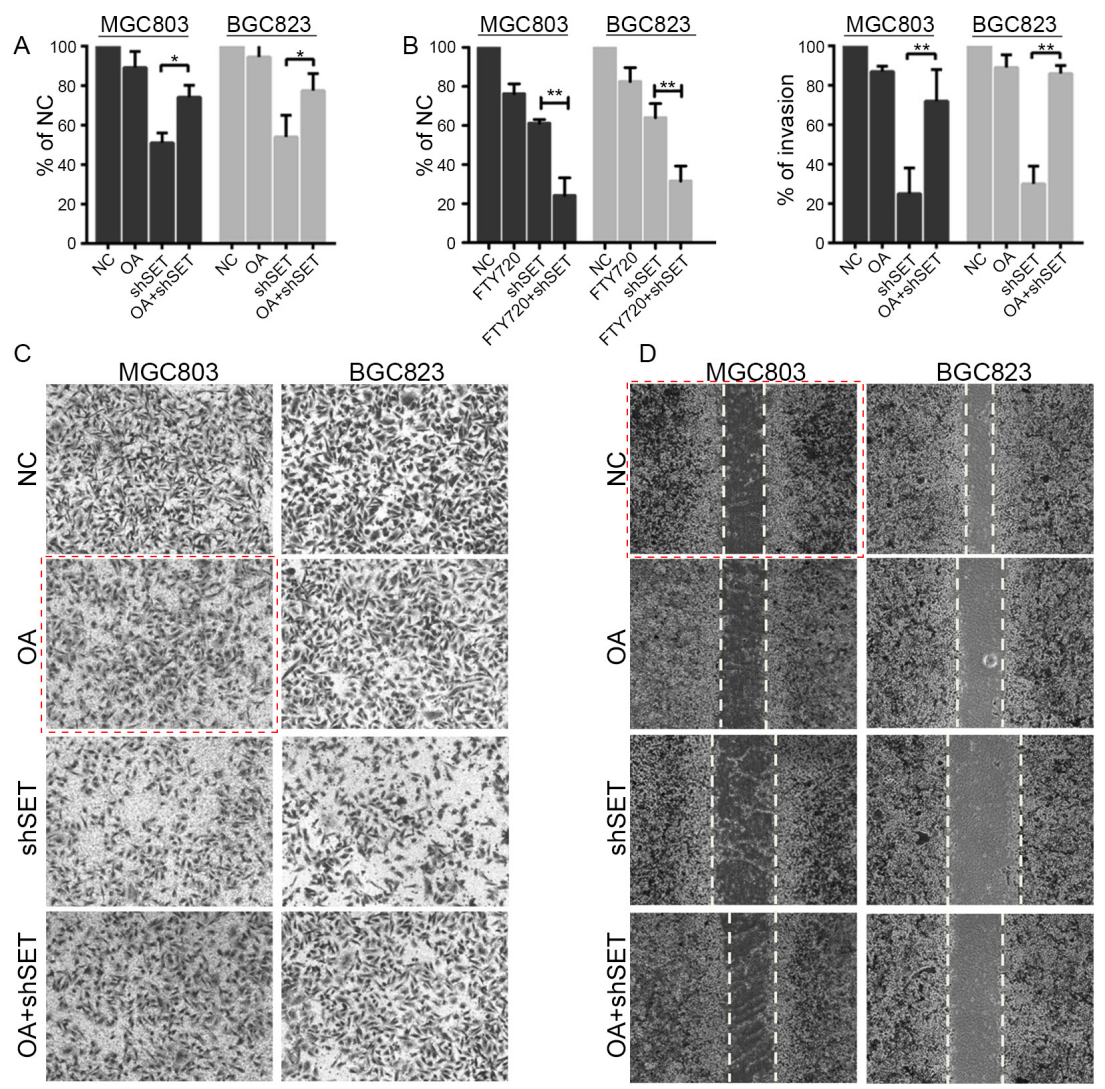
SET promotes proliferation and metastasis through inhibiting PP2A. (A) MGC803, or BGC823 cells were transfected with shSET (or NC), 6 h after transfection, cells were treated with or without 0.5 nM OA for 48 h, cell viability was evaluated by MTT assay. *p<0.05. (B) MGC803, or BGC823 cells were transfected with shSET (or NC), 6 h after transfection, cells were treated with or without 2.5 µM FTY720 for 48 h, cell viability was evaluated by MTT assay. **p<0.01. (C) MGC803 or BGC823 cells were transfected with shSET (or NC), 6 h after transfection, cells were treated with or without 0.5 nM OA for 48 h, cell invasive ability was detected by Matrigel Transwell assay. **p<0.01. (D) MGC803 or BGC823 cells were transfected with shSET (or NC), 6 h after transfection, cells were treated with or without 0.5 nM OA for 48 h, cell migration ability was detected by wound healing assay..

